# Insights into the constellating drivers of satiety impacting dietary patterns and lifestyle

**DOI:** 10.3389/fnut.2022.1002619

**Published:** 2022-09-20

**Authors:** Allah Rakha, Fakiha Mehak, Muhammad Asim Shabbir, Muhammad Arslan, Muhammad Modassar Ali Nawaz Ranjha, Waqar Ahmed, Claudia Terezia Socol, Alexandru Vasile Rusu, Abdo Hassoun, Rana Muhammad Aadil

**Affiliations:** ^1^National Institute of Food Science and Technology, University of Agriculture Faisalabad, Faisalabad, Pakistan; ^2^School of Food and Biological Engineering, Jiangsu University, Zhenjiang, China; ^3^Institute of Food Science and Nutrition, University of Sargodha, Sargodha, Pakistan; ^4^Department of Genetics, University of Oradea, Oradea, Romania; ^5^Life Science Institute, University of Agricultural Sciences and Veterinary Medicine Cluj-Napoca, Cluj-Napoca, Romania; ^6^Faculty of Animal Science and Biotechnology, University of Agricultural Sciences and Veterinary Medicine Cluj-Napoca, Cluj-Napoca, Romania; ^7^Univ. Littoral Côte d'Opale, UMRt 1158 BioEcoAgro, USC ANSES, INRAe, Univ. Artois, Univ. Lille, Univ. Picardie Jules Verne, Univ. Liège, Junia, F-62200, Boulogne-sur-Mer, France; ^8^Sustainable AgriFoodtech Innovation & Research (SAFIR), Arras, France

**Keywords:** appetite, food intake, food quality, diet, satiation, satiety

## Abstract

Food intake and body weight regulation are of special interest for meeting today's lifestyle essential requirements. Since balanced energy intake and expenditure are crucial for healthy living, high levels of energy intake are associated with obesity. Hence, regulation of energy intake occurs through short- and long-term signals as complex central and peripheral physiological signals control food intake. This work aims to explore and compile the main factors influencing satiating efficiency of foods by updating recent knowledge to point out new perspectives on the potential drivers of satiety interfering with food intake regulation. Human internal factors such as genetics, gender, age, nutritional status, gastrointestinal satiety signals, gut enzymes, gastric emptying rate, gut microbiota, individual behavioral response to foods, sleep and circadian rhythms are likely to be important in determining satiety. Besides, the external factors (environmental and behavioral) impacting satiety efficiency are highlighted. Based on mechanisms related to food consumption and dietary patterns several physical, physiological, and psychological factors affect satiety or satiation. A complex network of endocrine and neuroendocrine mechanisms controls the satiety pathways. In response to food intake and other behavioral cues, gut signals enable endocrine systems to target the brain. Intestinal and gastric signals interact with neural pathways in the central nervous system to halt eating or induce satiety. Moreover, complex food composition and structures result in considerable variation in satiety responses for different food groups. A better understanding of foods and factors impacting the efficiency of satiety could be helpful in making smart food choices and dietary recommendations for a healthy lifestyle based on updated scientific evidence.

## Introduction

The terms satiation and satiety are essential to understand the role of appetite in the regulation of food intake. Satiation is the feeling of fullness during an eating process, while satiety is the inhibition of hunger in response to eating (1). Hunger and satiety are involved in the maintenance of healthy body weight as energy intake and expenditure are mainly governed by the rate of gastric emptying as well as the metabolism of the nutrients. Energy balance is crucial to human survival and is dependent upon the amount of food consumed (2). Satiety not only determines the time elapsed between food ingestion at a meal and the next meal but also the prospective amount of food to be consumed at subsequent meals. The general population inherits the idea that foods with greater satiety are the ones that fill their stomach earlier. However, consumer perceptions are based on short-term satiety signals and orosensory learned indications. This area of consumer science is of particular interest in enhancing the knowledge and understanding of satiety perceptions among lay consumers (3).

The preparation and consumption of foods affect the mechanism of satiation and absorption of nutrients in the body. The feeling of hunger motivates the urge to eat. Thus, consuming food with superior satiating potential may help to achieve the desired dietary goals by decreasing overconsumption. From a nutritionist's perspective, satiety is helpful to prevent individuals from eating unhealthy foods (4). An unhealthy lifestyle that includes poor eating habits and unhealthy diet choices can lead to various chronic diseases including obesity, diabetes, dyslipidemia, hypertension, and cardiovascular diseases (CVDs) (5, 6). For example, obesity has become a prime cause of morbidity and mortality in many developed countries being a risk factor for several diseases (7). In the context of the prevalence of obesity among all age groups, it has become imperative to understand the satiating potential of foods as the energy intake of humans can be accurately predicted based on appetite sensations (8).

**Figure 1:**
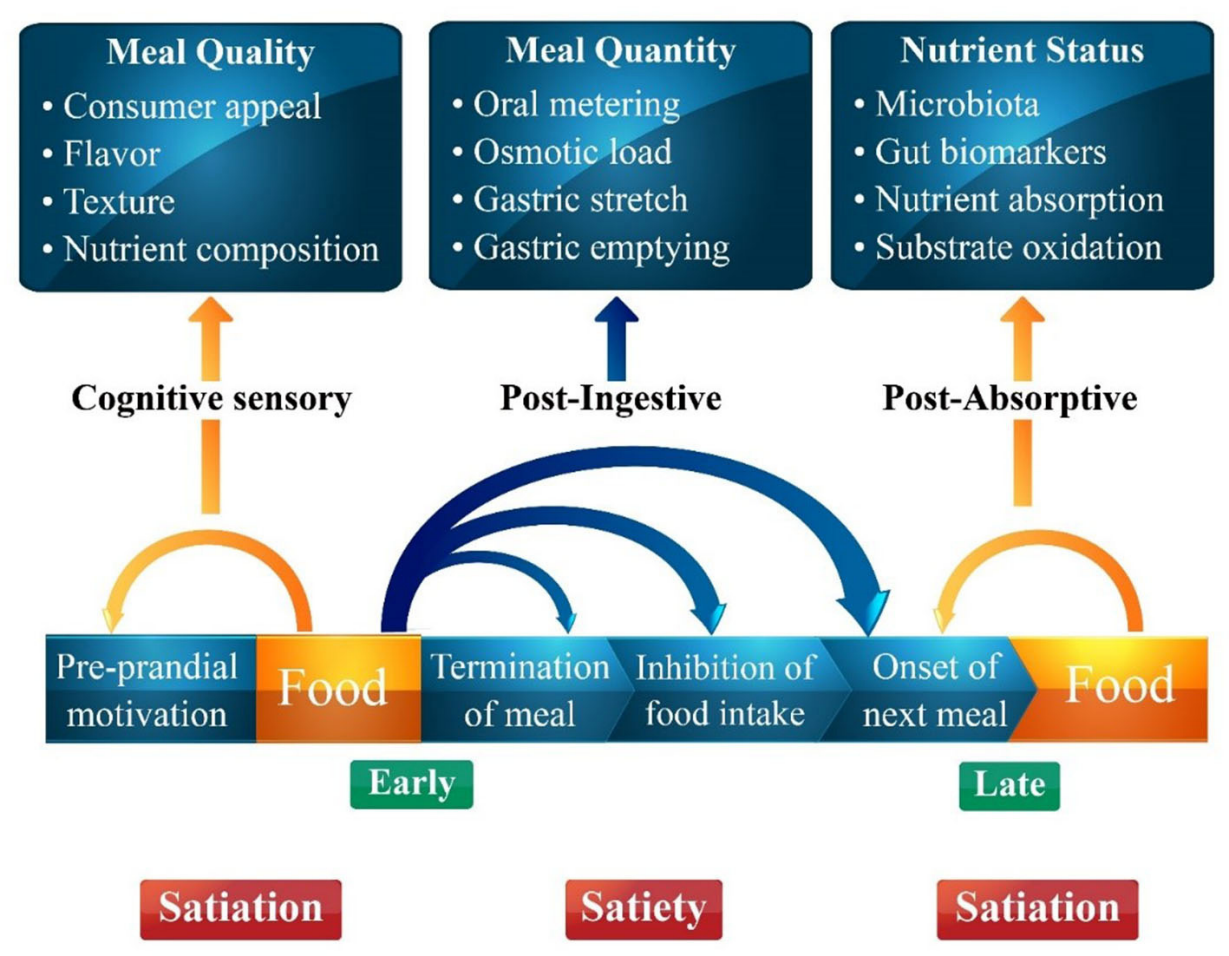
A schematic figure of the different components of the food before and after food intake determining satiation and satiety adapted from the satiety cascade model of Blundell and Gibbons.

Food intake is regulated by different factors including organoleptic properties, environmental factors, metabolic influences, physiological factors, social influences, psychological influences and food likes and dislikes (9). In the early stages, satiety is primarily influenced by orosensory and cognitive factors as previous experiences with taste, texture, flavor, aroma, and palatability drive the urge to eat. Similarly, meal quantity affects the digestion process, while post-meal absorption is affected by the nutrient status of the gut that in turn governs satiety. The interplay of different variables governing satiety and satiation is presented in Figure 1. (10). Food intake is compulsive action as mealtime continues, inhibitory influences from a variety of sources (sensory, gastrointestinal, hormonal, neurological, and cognitive) increase, bringing the meal to a halt. Satiation being a sophisticated inhibitory process integrates all these factors and brings a meal to an end (11). Meal size is determined by consumer satisfaction and many variables contribute to the inability to eat again until the following meal after one eating session has ended. The aspects of the “Satiety Cascade” were conceived as stimulatory and inhibitory impacts. The Satiety Cascade combines sensory, cognitive, post-ingestive, and post-absorptive components to reduce the desire to eat for a certain period. Satiety and satiation are strong processes for regulating total daily energy intake and expenditure because they include the suppression of hunger (12). Two foods with the same nutritional content may have distinct appetite-stimulating effects. This is because food consumption, aside from the metabolic effects of nutrients in the gastrointestinal system, contributes to the process of controlling appetite. The satiety cascade describes the signals that impact the primary appetite-control behaviors, such as food selection, satiation (the amount of food ingested within a meal), and satiety (the extent to which hunger and eating are suppressed between meals). The satiety cascade model predicts that the main drivers of satiation are early pre-ingestive signals from cognitive and sensory processes, and that cognitive, sensory, post-ingestive, and post-absorptive signals are combined to determine the experience of satiety, highlighting the integrative nature of satiety (13).

Although internal signaling systems (for the drive and suppression of eating) stimulate and inhibit eating behavior to regulate the internal environment (tissue needs, energy stores), sensory and external stimulation of food intake also plays a role as a hedonistic dimension of appetite. Likewise, the type of meal, timing, frequency, palatability, portion size and psychological factors also affect appetite (13). It is crucial to take into account the impacts of both individual and food variances for holistic studies that concentrate on the study of human satiety responses to foods. Multiple domains (physiology, psychology, eating and type of food) must be taken into account in order to comprehend the factors that influence perceived satiety, and there are significant individual variations that are in part influenced by external and internal factors (14). Therefore, it is essential to analyze the physiological as well as behavioral aspects to completely understand the role of satiety and satiation in individual eating behavior. Consequently, we have focused here on the methods of satiety measurement, factors affecting satiety, and variation in satiety response among different food groups. Most of the previous reviews have individually discussed the role of body composition (15), sensory specific food cues (16), taste perceptions (17), gut microbiota (18), energy density (19), physical properties of food (20), and intestinal hormone receptor (21) modulated effects about food intake, satiety, or satiation. However, a recent appraisal of the strength of evidence for external or internal factors influencing appetite has not been reviewed. What is also not clear from the work presented so far is how critical it is to integrate different internal or external factors either food-related and personal factors controlling food intake to maximize the individual potential for improved satiation or satiety. This review aims to gather relevant existing knowledge on food intake regulation and satiety considering the role of the most promising factors involved in lowering the energy intake or controlling food intake that ultimately helps in obesity reduction or other chronic diseases. Core evidence for the satiating potential of different isocaloric foods is also carefully summarized in this study. The role of satiety hormones and modulation of different orosensory cues along with the effect of food texture expected satiety on portion sizes, age, gender, and the response of different functional foods from various food groups on ingestion in delaying the appetite sensations has been discussed. Moreover the interplay of bioactive ingredients and functional foods in relation to appetite control, satiety or body weight reduction has been considered.

## Measurements of subjective satiety

Satiety is a subjective measure of appetite as people feel hunger differently. Various methods have been used for the measurement of satiety owing to the difference in standardizing the test instructions to participants (22) and the lack of standardized protocols (22). The major problem in the assessment of satiety is attributed to the non-uniform perception of satiety sensations among different individuals. Purposely, assessment of satiation and satiety is normally carried through either of the following methods: subjective appetite rating, *ad libitum* intake, and physiological measurements (23).

Previously, many other scales have been employed for the measurement of satiety such as a seven-point scale (24), a labeled magnitude scale (25), and a triangle rating scale (26). Nevertheless, VAS remains the most frequently used scale in subjective measures of satiety. Earlier, a satiety index was developed by Holt et al. (27) in Australia to compare the satiety value of different foods using a number or value. In this context, a VAS was used to assess the subjective response of the participants to different food items. The data was recorded by taking the appetite ratings before and after 120 min of food ingestion. The satiety response curves of the test foods were compared with the reference food (white bread) (27). The satiety rating of the bread was assigned a score of 100, while the satiating potential of all other foods was determined based on ranking against reference bread as illustrated below (Equation 1).


(1) $$\begin{array}{l} Satiety\;index\;\left(\%\right)=\frac{Sample\;score\;}{Reference\;bread\;score}\times 100 \end{array}$$

Subjective ratings of appetite have been conducted using a visual analog scale (VAS). The scale comprises a scale that is either 100 or 150 mm in length. The subjects in question rate their feeling of appetite by placing a mark on the scale in response to different questions posed, whereas the distance from left to the marked point is recorded to calculate the satiety value. A graph is developed by taking the post-meal consumption appetite readings after every 2–3 h interval. VAS is a reliable and valid tool for satiety measurement under controlled settings (28). The following questions form the basis of the VAS scale assessment including (1) How hungry do you feel? (2) How much food do you think you could eat? (3) How strong is your desire to eat? (4) How full do you feel? usually asked to complete the assessment (28). These measurements offer insightful data on sensations that are challenging to record using other techniques. Pen and paper were used to administer VAS in the past since it was quick and simple. However, as each line must be physically measured and entered into a database one at a time, the pen-and-paper technique of data gathering is frequently time-consuming and subject to human error. Portable handheld computers have been created to electronically administer appetite scales, solving the issues with pen and paper (Electronic Appetite Ratings System or EARS). The laboratory test meal approach has been used in some significant experimental investigations to support the validity and reliability of VAS as a measure of the intensity of the incentive to eat (29).

The relationship between energy and the satiety score of different foods can be a useful addition to nutrition facts tables on the food labels. Similarly, a satiety quotient (SQ) describes the satiating efficiency of foods and the amount of energy consumed. The SQ was computed by dividing the difference between pre- and post-eating episode assessments of motivation to eat (pre minus post) by the energy content intake during the episode of eating. Subsequently, the SQ can be calculated using the following expression (30) (Equation 2).


(2) $$\begin{array}{l} \text{Satiety Quotient}=\\ \frac{\text{Pre}-\text{eating episode rating }\left(\text{mm}\right)\;-\text{ Post eating episode rating }\left(\text{mm}\right)}{\text{The energy content of the test meal }\left(\text{kcal}.\right)} \end{array}$$

However, since other factors affect fullness and satiation, subjective sensations do not give a complete picture of appetite control and calorie intake. This method also enables the calculation of the satiety quotient about the energy/weight content of the meal offered, allowing for the measurement of subjective appetite about the quantity of energy consumed. However, the results of such studies may be found to be more meaningful when the eating pattern and study schedule resemble in terms of eating duration (3–4 h) that is followed normally by participants (29). Following the start of preload ingestion, a typical *ad libitum* test meal made up of well-known, easily accessible foods and water are usually provided. Then subjects allowed for a specific window of time to consume till they are satisfied and are allowed to ask for more food if desired. The idea behind the method is that interventions that increase satiety will cause people to consume less during a typical meal and vice versa (22). Since hunger is a definite factor in determining food intake, the participants must be in similar appetite states while evaluating energy intake. Before being served an *ad libitum* meal, participants' access to food and beverages should be restricted to maintain a consistent level of hunger among participants and across situations (29).

Integrating physiological measures to record changes in satiety indicators in the postmeal interval can improve sensitivity and discrimination in satiety responses to various treatments. Postmeal phenomena such as changes in gastric emptying rate, circulating levels of certain gastrointestinal hormones such as glucagon-like peptide-1 (GLP-1), peptide tyrosine tyrosine (PYY), cholecystokinin (CCK), and polypeptide-P (PP), and suppression of ghrelin are more pertinent when exploring satiety (22, 29). Studies on these physiological biomarkers provide evidence of their role in regulating appetite and calorie intake. The practicality of quantifying these peptides has several challenges. Since the peptides break down so quickly, it is necessary to implement regular processes to stop this. Thus, these postprandial investigations detecting physiological indicators associated with appetite are very challenging and expensive to conduct.

Like other electronic tools, near-infrared (NIR) spectroscopy, a potent optical analytical technique, is effective for the non-destructive and label-free evaluation of biochemical, molecular, and structural information in biological tissues, including human tissues. Human tissue has biomarkers that reveal information about metabolic health and body composition, such as the proportion of lean to fat muscle tissue and body fat. A hand-held portable NIR equipment was tested by Ni et al. (31) for its capacity to capture the spectra of human tissues (arm, face, ear, mouth, and wrist) and to predict satiation, fullness, and food intake in participants from a sensory investigation. Results suggested that it would be able to evaluate the complicated interactions between humans and food by using the NIR spectra of tissues as a proxy. Variations in the cross-validation statistics were also noted, and they were strongly influenced by the type of tissue examined, metabolism, and body composition. A variety of electronic devices have been approved for use in assessing appetite for hunger or fullness as recent models made possible by their economic and other practical advantages.

A variety of factors might affect the feeling of fullness. So for a true assessment of meal termination, only one component should be permitted to change at once. It is rather unclear how similar studies of a kind may exist because study designs can vary greatly. Comparisons can be performed if the study is planned to take into account the aforementioned factors, although caution should be used when approaches diverge significantly. Appetite measurements should include a comprehensive collection of measurement techniques that allow for the evaluation of the potency of the motivation to eat, key food choices, and hedonic processes that modify the homeostatic system (29).

## Factors affecting satiety

**Figure 2:**
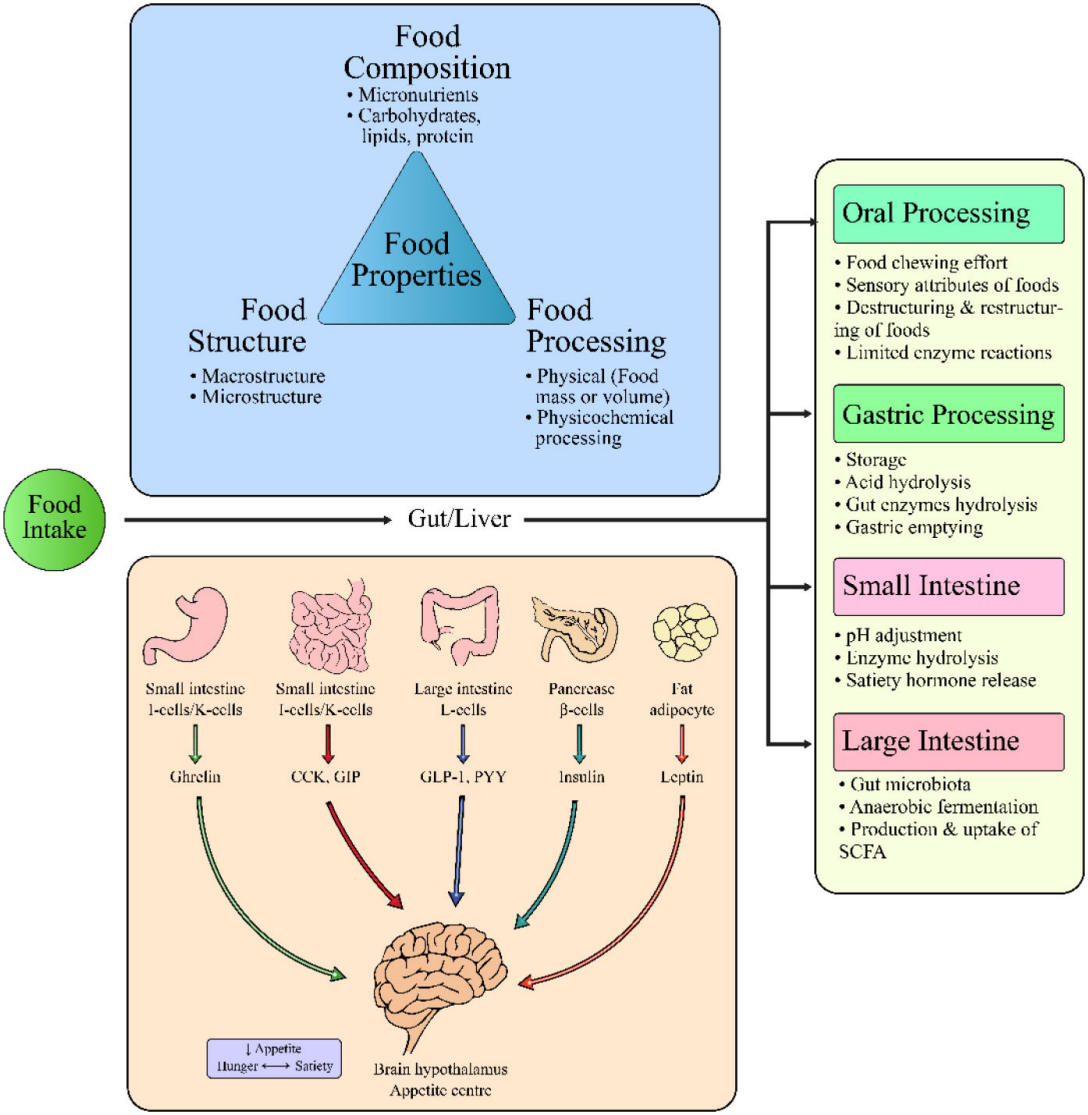
Interrelation of food properties and internal factors controlling food intake, satiation or satiety.

**Figure 3:**
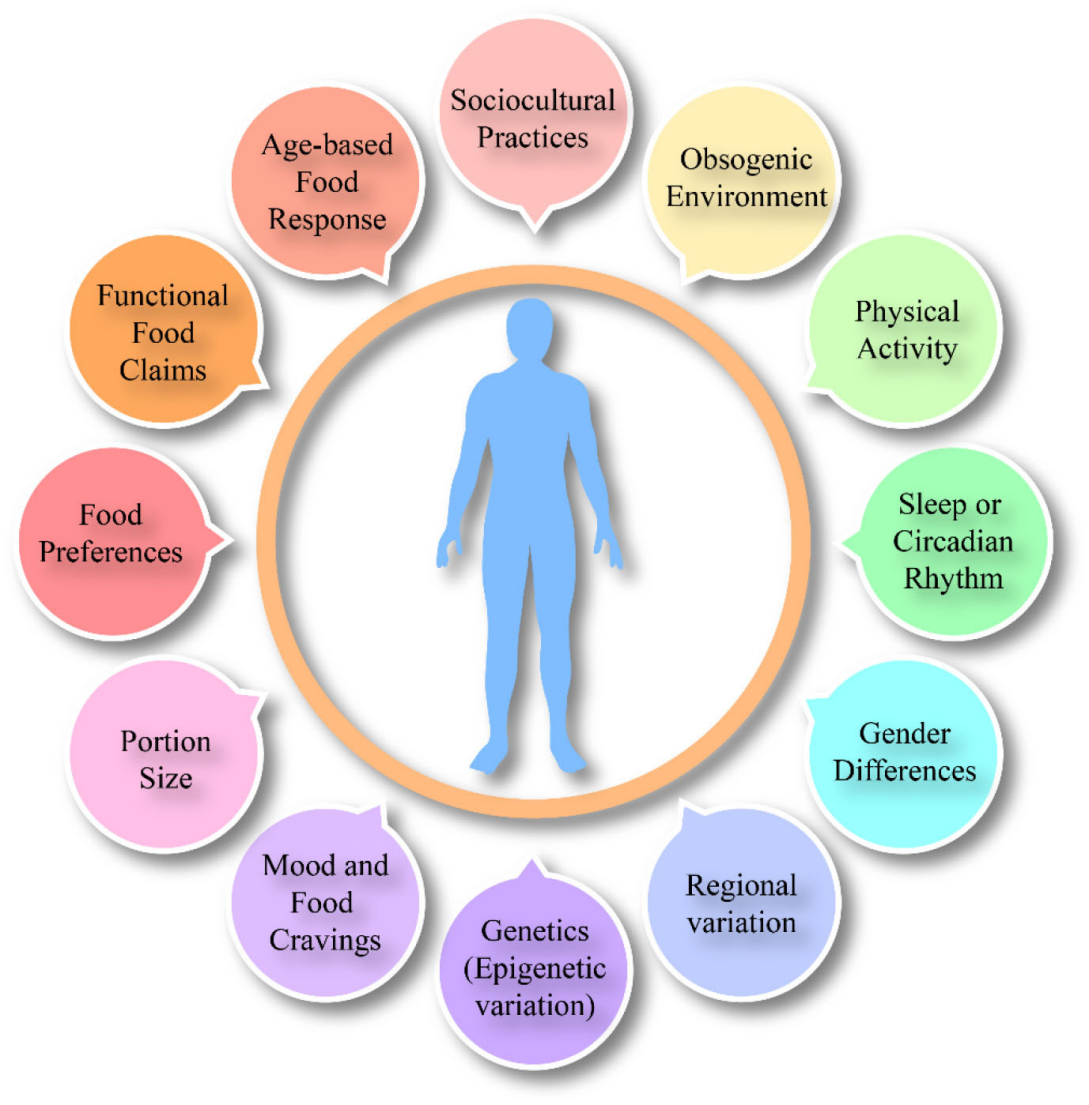
Overall external factors affecting food intake, satiation and satiety.

The influence of different internal factors (Figure 2) and external factors (Figure 3) on appetite, satiety and satiation have been discussed that possibly affect food intake. Although the literature on these external or internal variables and satiety is complicated yet all these factors have been potentially studied with eating behaviors.

### Personal factors

#### Physiological

The gut is the largest organ of the body for hormone production as well as the presence of various enterocytes (32). Initially, satiation is influenced by the stomach distension as mechanoreceptors send signals to the hypothalamus *via* the vagus nerve located on gastric distension. When food passes through the gut, multiple peptides are released from the specific enterocytes of the stomach and small intestine including cholecystokinin (CCK), neurotensin, gastrin, glucagon, somatostatin, peptide YY (PYY), bombesin, and glucagon-like peptide-1 (GLP-1) (33). Hence, the physiology of food intake regulation involves precise coordination between neuronal and hormonal signals. Among them, ghrelin (orexigenic hormone) is the only hormone released from the oxyntic glands of the stomach which triggers appetite and favors feeding by enhancing the incentive and hedonic response to food-related cues (34). Other hormonal signals released from either of the upper or lower intestinal tracts involve leptin, PYY, CCK, and GLP-1 are responsible to suppress hunger (29, 35). The GLP-1 (anorexigenic hormone) releases from the small intestine in response to contact of glucose with L-cells, causing a drop in hunger. Thus, slow digestion of food can sustain prolonged intestinal contact with glucose, thereby improving satiety. Likewise, CCK releases from the small intestine in response to the fat and protein contents of the food being ingested to suppress the appetite as soon as the signal reaches the nucleus solitarius tractus (central nervous system) through the vagus nerve (36). The long-term food intake is regulated by the leptin secreted from adipose tissues, thereby maintaining energy balance (33).

#### Gut microbiota

The interplay between gut microbiota, satiety hormones and energy intake along with underlying mechanisms have been well studied. Often, obese people tend to be insulin resistant, and modifications in host bacterial interactions with dietary intake can be beneficial in suppressing the appetite (37). Enteroendocrine cells generate intestinal hormones such as CCK, GLP-1, and PYY, which play an important function as signaling systems. The stomach and various brain areas have been found to contain receptors for these hormones, emphasizing the gut-brain relationship in satiation mechanisms. Diet can modulate the intestinal microbiota, which interacts with enteroendocrine cells, by delivering certain nutrients that cause changes in the gut ecology (dysbiosis) due to hyperphagia. As a result, macronutrients may activate the microbiota-gut-brain axis *via* mechanisms such as particular nutrient-sensing receptors in enteroendocrine cells that cause hormone release. This results in a reduction in appetite or an increase in energy expenditure (38). In this regard, prebiotics has demonstrated their efficiency by increasing the expression of anorexigenic hormone (GLP-1) which in turn acts on the brain to signal hunger or satiety. The proposed mechanism is considered to reduce the gastrointestinal transit time by acting as an ileal break (39). Likewise, the production of short-chain fatty acids (SCFA) by the gut bacteria while metabolizing non-digestible carbohydrates has been shown to upregulate gene expression of proglucagon, the precursor to GLP-1 and PYY43 in the intestinal tract (39). This phenomenon leads to increased satiety and decreased food intake after the meal. Short-chain fatty acids can trigger intestinal gluconeogenesis through a cyclic adenosine monophosphate-dependent mechanism (40) which has positive effects on glucose and energy balance. Propionate, for example, is an energy source for epithelial cells, but it is also transported to the liver, where it contributes to gluconeogenesis. Because of its interaction with gut receptors, it's becoming more well recognized as a key component in satiety signaling (41). Likewise, ghrelin levels have shown negative correlations with *Lactobacillus, Bifidobacterium, Blautia coccoides*, and *Eubacterium rectale*, whereas the inverse was observed with *Prevotella* and *Bacteroides* (42). This signifies the role of gut microbiota in the satiety regulation and interaction with ghrelin and leptin. Moreover, butyrate production in animal models has been associated with serotonin levels which are an important neurotransmitter in the brain and gut, involved in the regulation of satiety and body weight (42). It is not clear, if gut microbiome composition changes are driven by a decrease in leptin action, as a consequence of hyperphagia, physiological modifications associated with obesity, or other leptin actions independent of food intake and adiposity. Moreover, the leptin signaling pathway related to leptin receptor (LEPR) extracellular domain mutation suggests its role against gut pathogens and it seems that leptin signaling may also have a role in modulating gut bacterial microflora, independently of food intake, by regulating gut antimicrobial peptides expression (43–45). Moreover, gut microbiota might be associated with leptin resistance, which is in general developed in obesity, throughout interfering hypothalamic and brainstem neural processes, involved in feeding and energy balance control (46). All of these suggest that gut microbiota modulation could be a novel therapeutic target in obesity focusing on leptin signaling (47). Besides, the prebiotic effect related to gut microbiota modulation refers to a higher leptin sensitivity and glucose tolerance, and lower oxidative stress and inflammation (48).

Although clinical trials have shown alteration in human gut microbiota after consumption of maize, whole grain wheat, and barley, there is no functional link between fullness and gut microbiota due to a lack of valid satiety assessments (18). Decreased sensation of hunger on *ad libitum* lunch intake in healthy young men by consuming wholegrain rye may be partly mediated by colonic fermentation as *in vitro* fermentation profile of rye kernel also confirmed SCFA production after 24-h of fermentation study (49). Likewise, when compared to the breakfast of refined wheat bread, rye kernels improved satiety most substantially, both immediately and in the face of a second meal, as evidenced by lower energy intake at lunch and self-reported VAS ratings. The researchers hypothesized that greater microbial fermentation or increased fermentation metabolites could be observed, as seen by higher breath hydrogen levels after eating whole grain rye bread than refined grain wheat bread. These fermentation products may help with glucose management and satiety by delaying the release of ghrelin, the hunger hormone (50).

#### Sociocultural

Mostly, physiological aspects of appetite regulation are studied, however, it is imperative to include social dimensions of satiety for a better understanding of the underlying phenomenon. Variations in specific cultural patterns of cuisines and food intake affect satiation and satiety and are primarily dependent on meal size. Furthermore, consuming food with other persons may increase the intake by up to 44% and it tends to increase successively in the presence of more people. Therefore, the company of the eaters such as family, spouses, friends, and colleagues at mealtime influences the energy intake. Moreover, eating foods under different conditions and the nature of the companions control the energy intake, as obese individuals tend to consume more food in the presence of obese in contrast to non-obese individuals (51, 52). Social isolation, poverty, and loneliness are the other predominant factors regulating food intake, thereby in turn appetite sensations. Other determinants of food choice include socio-economic factors, media literacy level, social inequality, family dimension, health, ease of access, occupation, taste, food preferences, knowledge, peers, friends, parental education; nutritional quality of food, cooking skills, life course, past experiences, ethnic customs as well as past eating habits (53). Physical conditioning and emotional reactions to the social setting in which eating occurs can also affect how you feel about being full. Parents substantially shape the context in which children encounter food by regulating, encouraging, restricting, and rewarding food (54). Although our determinants for food choices are greatly influenced by biology. Though the biological factors that regulate food intake can be modified by disease conditions, experience, or learning. Other social and environmental influences also affect the relationships between the person and their dietary choices relate to familiarity and learnt safety, conditioned food preferences, and conditioned satiety (55).

#### Psychological

Satiety is a complex phenomenon and must be interpreted from both metabolic and behavioral perspectives. The psychobiological dimensions of satiety involve three events i.e., hunger perception, food cravings, and hedonic sensations. Consumption of food triggers various physiological events that in turn control the neurochemical activity of the brain which represents the desire to eat and willingness to refrain from eating (56). Psychological aspects that govern meal-by-meal appetite make it necessary to highlight their impact as a conditioning factor of satiety regulation.

Individuals on a weight-loss dietary regimen have demonstrated that appetite is merely linked with distinct psychological phenomena such as feelings of deprivation, increased reinforcing value of food, cravings, increased subjective appeal of energy-dense foods as well as an increased central nervous system (CNS) reward system feedback to calorie-rich foods. Regulation of food intake by maintaining homeostasis between reward and inhibitory controls of food cues plays an important role in conditional eating and subsequent appetite responses (57). Considering sensory-specific satiety, concepts of food acceptance and rejection play an important role in determining personal eating patterns. Likewise, cognition affects the eating process as conditioning to specific food cues can alter the food intake pattern. Learning about different foods and developing likes and dislikes throughout life are associated with certain conditioned and unconditioned reflexes, affecting behavioral eating patterns. Similarly, foods consumed before exercise can improve cognitive functioning and positively influence the mood of people with improved appetite control (58) as a result of improved insulin sensitivity and glucose response after a meal.

#### Environmental factors

Although overconsumption norms are prevalent in our society, studies suggest that portion size directly impacts food intake regardless of hunger level and taste preferences. Along with different consumption patterns and utensil size illusions, environmental interferences such as watching television (T.V.) or listening to music can affect both food selection and intake. Besides these dynamics, watching TV during eating is the most important factor affecting satiation and satiety as it directly influences energy intake. Reportedly, viewing TV can significantly impact appetite ratings with an increased food or energy intake (59). Likewise, a study was designed to reveal the outcomes of watching TV while eating using two energy-dense foods. Participants were randomized into two groups receiving macaroni and cheese or pizza as a test meal. While watching TV programs of their choice, readings for energy intake, hunger, satiety, and palatability were taken. Results revealed 71% higher energy consumption from macaroni and cheese and 36% from pizza (60). Similarly, another study concluded that consuming a meal while watching TV not only enhanced energy intake at mealtime but also affected the normal mealtime satiation or satiety followed by reduced satiety signals from previously consumed foods (61). People usually eat those foods with enjoyment that they like in contrast to the less-liked ones, as they experience more satisfaction, pleasure, and satiety after consuming the meals of their liking (62). Environmental factors that may influence food intake and food selection include the size of the portion, the presence of other people, the location and the time of consumption. More specifically, it has been demonstrated that the color of the plate ware, the packaging, and the atmosphere all have an impact on food consumption (63). Consumers may be able to prevent overconsumption by being aware of environmental cues such as illusions, distractions, portion sizes, and variety. As appetite reflects the expression of the urge to eat and the behavior that is directed toward the intake of food and drink items readily available in the environment. Therefore, environmental or contextual factors that may be implicated in meal termination should also be taken into consideration (29).

#### Gender differences

Gender difference affects food intake regulation, appetite control, and management of healthy body weight. Females are easily satiated during eating as compared to males due to the involvement of certain hormonal and neuronal activation (64). The impact of gender difference on hunger scores revealed that women ate the given amount of isocaloric *ad libitum* food satisfactorily while men did not satiate easily and consumed significantly more (65). The difference in body composition of males and females is an important contributor to the variable food/energy intake. It is pertinent to mention that women possess significantly more body fat when compared to men, hence having more leptin levels in the body (66). The leptin is secreted from adipose tissues and promotes satiety by acting on the hypothalamus. Owing to higher adiposity in females, leptin secretion is relatively more as compared to males, which results in declined food intake and energy expenditures (67). When the fat cells increase in number, leptin levels increase proportionally, and then bind to LEPR in the brain, which sends signals to inhibit food intake and increase energy expenditure. No matter how, when caloric intake exceeds energy expenditure (positive energy balance) is sustained for critical periods, weight gain occurs (68). The majority of obese people have hyperleptinemia and do not respond to leptin therapy, showing leptin resistance and casting doubt on leptin's function as a human energy balance regulator. Chrysafi et al. (69) showed that long-term leptin treatment lowers fat mass and body weight and transiently modifies circulating free fatty acids in lean slightly hyperleptinemia people, but short-term leptin administration alters food intake during refeeding after fasting.

#### Age differences

Age is an important element that regulates satiating efficiency of foods since sensory-specific satiety declines in old age due to age-associated changes (increases in intensity discrimination) for taste and smells, reducing energy intake. Therefore, old-aged people can easily get satiated and become leaner with increasing age (70). In this perspective, a study was conducted to assess the age mediated difference in sensory-specific satiety. For this purpose, adolescents, young, older adults, and elderly persons were recruited for the study. The results obtained showed distinct differences in sensory-specific satiety among adolescents and the elderly. This explains the limited food choices in elderly persons owing to a decrease in food pleasantness which may lead to serious health threats (71).

#### Effect of chewing

The impact of chewing on satiety is evident from the fact that people usually chew less if food is more palatable, hence promoting food intake. Thus, chewing food can alter the eating rate and digestion. The chewing rate is associated with satiety and is usually higher for mixed meals when compared to single food (72). The mastication of almonds resulted in a decline in GLP-1 (orexigenic hormone) and an increased fullness after 40 chews as against 25 chews (73). Chewing may enhance or reduce hunger and relative food intake through gut hormone response modification. For example, chewing gum has enhanced the feeling of satiety in obese as well as healthy weight women (74). Moreover, video recordings of chewing gum at different frequencies also showed variations in chewing (75). The findings of the study revealed that the rate of chewing particularly depends on the type of food being consumed. Increased rate of chewing in each mouthful for sustained meal duration had shown a decline in food intake (76). Similarly, a decreased snack intake was observed after 2 h of having lunch with prolonged chewing. Thus, a higher number of chews promote slow eating and may help obese people with less caloric intake (77). Furthermore, the rate of food biting has been associated with energy intake, with a slower rate being more useful to decrease food intake. Interestingly, obese people usually take large bites as compared to lean individuals, affecting the rate of food ingestion (swallowing) and successive increase in energy (78). In fact, recent research showed that extending the time between chewing and swallowing reduces food consumption and boosts fullness. Although it has been demonstrated that delaying eating can prevent weight gain in children and adolescents, it is unclear if slowing eating by increasing the number of masticatory cycles or lowering the mastication rate is a practical way to support weight management (79).

#### Physical activity

Physical activity is another important parameter governing appetite by improving the sensitivity to physiological responses regulating satiety. Purposely, the effect of physical activity on appetite along with satiety scores was assessed in obese women (average BMI of ?37) after 20 min of brisk walking. The results suggested that even moderate physical activity is vital in modulating the role of postprandial peptides (insulin and leptin) in the short-term regulation of food intake (80). Therefore, consistent physical activity can improve appetite control by improving satiety signaling. However, specific actions, intensity, and duration of exercise can affect physiological elements of satiety, which also varies from person to person based on individual physiology (81). Hence, regular exercise has a strong potential to control appetite and satiety (82). In general, leptin is overexpressed in obese individuals, and its altered expression leads to leptin resistance, which implies mechanisms interfering with leptin's ability to reach targeted cells due to decreased LEPR expression or altered signaling. Genetic variations in the LEP gene can modulate its circulating levels and interfere with various pathophysiological processes (47). In this context, in the last few years, increased obesity prevalence as a consequence of a sedentary lifestyle and low physical activity has been linked to systemic, chronic low-grade inflammation processes through adipocyte-secreted hormones (adiponectin, leptin, resistin, and ghrelin), growth factors and proinflammatory cytokines (83). Studies on rodents and humans provide evidence that the majority of exercise induced favorable effects on obesity are linked to lower leptin levels and improve leptin resistance. For instance, in obese adolescent girls, a 12-week combined resistance and aerobic exercise training efficiently reduced body weight, waist circumference, and serum leptin levels, hence reducing central leptin resistance (84). Combining resistance and aerobic exercise training also improved the cardiometabolic indicators of older men with obesity along with a reduction in leptin levels (85). High-intensity interval training, other than combined training, also decreased body fat and inflammation in obese postmenopausal women, along with a significant drop in leptin levels (86).

#### Sleep and circadian rhythms

Sleep is also another important factor for appetite control and laboratory studies demonstrated that sleep deprivation impairs insulin sensitivity and glucose disposal throughout the body. Individuals recruited in a study trial stated that sleep restriction lowered participant-perceived fullness or satiety as well as suppressed the postprandial lipemic response and decreased satiety (87). As the SQ governs the extent to which a meal can minimize subjective appetite sensations, SQ in response to a standardized meal was assessed in overweight or obese men according to sleep duration for a later bedtime and poor sleep quality in association with energy intake. Results revealed that short-duration sleepers had a lower mean SQ than recommended sleep duration sleepers without impacting overall energy intake (88). Interestingly, another study found greater total food-craving scores in subjects in association with increased daytime sleepiness, when participants were assessed by a 7-day sleep-hunger-satiety diary (89). A lot of people who live at home don't get enough sleep. When volunteers were experimentally sleep-restricted but had unlimited access to food, they consumed more calories than when they are not sleep-restricted; these calorie increases are often observed in post-dinner snack patterns (90, 91).

Energy consumption during inappropriate circadian periods is one potential reason for negative health effects during circadian disturbance and inadequate sleep. Lab investigations have shown that when people are given meals during the circadian night (when melatonin levels are high), they have a lower energy response than when they are given meals during the day along with impaired glucose tolerance (92, 93). There is compelling evidence that energy consumption later in the day may contribute to ill health during both circadian disturbance and inadequate sleep. Though circadian rhythm was not altered in conditions with sleep episodes lasting < 6 h per night (i.e., chronic sleep restriction) in which participants were given a diet designed to meet caloric needs (94). This showed that circadian timing, rather than sleep limitation, might play a significant role in hunger patterns. However, it is uncertain how several days of energy consumption during the circadian evening and night, as well as at a period when melatonin concentrations increase favoring sleep (i.e., chronic circadian disruption) may affect hunger, appetite, and food choices (95).

The integration of internal circadian rhythms and external cues such as the light-dark cycle and dietary composition is critical for survival and requires temporal partitioning of daily food intake. These internal and extrinsic variables are interrelated, with circadian rhythm misalignment encouraging body weight increase and calorie-dense diet intake increasing the risk of obesity and blunting circadian rhythms (96).

#### Genetics

Mealtime, the quantity of food consumed, and food preference are all influenced by a complex interaction of physiological, psychological, and social interactions along with genetic variables (97). Heritability and linkage analysis of individual food-consuming behavior measured by the three-factor questionnaire (TFQ) provides evidence that eating behavior traits are heritable. A growing body of evidence links hedonic signaling to the obesity epidemic in addition to the role of the hypothalamus and hindbrain in homeostatic food intake and satiety. The hippocampus is particularly rich in genes associated with human genome-wide association study (GWAS) obesity loci. A high-fat diet and obesity have frequently been associated with hippocampal atrophy, which may potentially affect responses to taste. The hippocampus may help regulate meal size (98, 99). The nucleus accumbens has been studied for obesity therapy because it can impact food intake pathways (100). In the insula and substantia nigra, areas implicated in addiction, motivation, and reward-seeking behavior, a recent study found substantial gene expression enrichment of top obesity/BMI-associated loci (101). Depending upon the nutritional status for eliciting the act of producing a satiety response, the hypothalamus communicates with the insula (102).

The fat mass and obesity-associated gene (103) is one of the most important obesity-associated genes discovered using GWAS. In the first intron of the FTO gene, several variations have been discovered that are linked to increased calorie ingesting, body fat, weight, and other adiposity measurements (104). About a 1.7-fold increase in obesity risk has been observed in patients that are homozygous for the “A” allele relative to the low-risk “T” allele owing to one of the best-studied FTO rs9939609 variants (105). Besides, postprandial appetite reduction in subjects noted that are homozygous for the A allele because of dysregulated circulating levels of acyl-ghrelin, suggesting that variations in FTO may change the action of ghrelin, the hunger-promoting gut hormone (e.g., reduced satiety response) (106).

The satiety pathway is usually well controlled. The LEPR and the melanocortin-4-receptor (MC4R) genes are two of the most investigated genes expressed in the brain, revealing biological mechanisms not yet fully elucidated. Several single nucleotide variations in LEPR have been linked to severe obesity, including Lys109Arg and Gln223Arg. According to recent studies, roughly 7% of the general population as well as obese persons accounting for more than 10% of the population had a coding variation in MC4R. About 20% of single-nucleotide variants in the MC4R gene have been projected to be pathogenic or likely pathogenic, emphasizing MC4R's prevalence in monogenic obesity (107).

In leptin-deficient people, leptin replacement can enhance satiety and help them lose weight. Leptin stimulates the production of a melanocyte-stimulating hormone (a-MSH), which induces satiety. Eating habit has also been connected to GAD (glutamic acid decarboxylase), which converts glutamate to GABA (g-aminobutyric acid), a brain inhibitory neurotransmitter. Disinhibition and disordered food consumption, notably higher carbohydrate intake in women, have been linked to two specific GAD variations, rs7908975 and rs992990 (97). Even though in the last years, several complex mechanisms related to energy regulation and obesity have been proposed, further studies are needed for a better understanding of interactions between genetic, environmental, and lifestyle factors that contribute to obesity (108).

#### Mood and food cravings

Food desire is considered to be one of the main elements influencing eating behavior, along with hunger, which is brought on by food deprivation or fasting. Although healthy adults with typical eating habits experience food cravings, research indicates that intense food cravings may be a risk factor for binge eating, which may lead to weight gain and obesity. Food cravings are viewed as a motivational state that is conditioned in response to sensory, environmental, or interceptive inputs (109). Moreover, Reents et al. (110) used a food cue-reactivity paradigm on normal-weight females to more thoroughly investigate these impacts on momentary food seeking. The states of food deprivation (hunger vs. fullness) and mood (negative vs. neutral) were changed systematically. In comparison to stated states, the self-rated craving was much higher when one felt hungry. Additionally, high-calorie foods reduced cravings in a neutral mood; hence, people who were hungry or satisfied preferred savory food and sweet food, respectively. This distinction between the effects of savory and sweet foods was not seen in a depressive mood. In conclusion, hunger has a significant impact on food cravings, which are further influenced by emotional state.

#### Gut enzymes and gastric emptying

Enzymes can greatly contribute to digestion-induced changes in the food structure. Since gastric cells secrete hydrochloric acid in reaction to food entering the stomach, the stomach has a strongly acidic environment with a pH of roughly two. The stomach secretes two enzymes that help break down proteins (pepsin) and lipids (gastric lipase). Depending on the rate of mixing and acid production, salivary amylase probably continues to work on carbs in the stomach for some time (111). One of the key factors in food disintegration in the stomach is the hydrolysis of proteins by pepsin. Several food particle-specific characteristics, including the solids content, density, internal tortuosity, surface-to-volume ratio, and porosity, affect the diffusion of pepsin into food particles (112). Different fluorescein isothiocyanate pepsin diffusion coefficients of two egg white gels were reported with the same protein concentration (10 wt%) as structures induced at pH 5 or 9 were found to vary more. The pH 5 gel displayed a greater diffusion coefficient than the pH 9 gel due to the pH 5 gel's more loose, spatially heterogeneous protein matrix and homogeneous microstructure (113). To produce free fatty acids and 1,2-diacylglycerols, gastric lipases preferentially hydrolyze the sn-three position of triacylglycerols. Triacylglycerols that have been consumed by healthy persons undergo 10 to 30% lipolysis during stomach passage (112). Food macromolecules are broken down in the small intestine, which functions as an enzyme bioreactor, by the hydrolytic processes of the carbohydrates, proteases, peptidases and lipases. Low molecular weight hydrolysis products diffuse out and are then absorbed into the bloodstream. Additionally, bile acids are released from the gall bladder duct and assist to emulsify lipids which facilitates breakdown by pancreatic lipase (111). The protein conformation, the presence of cross-linkages between protein chains, binding metals or polyphenols, the particle size, and the presence of anti-nutritional factors like trypsin and chymotrypsin inhibitors also have an impact on how food proteins are digested. Additionally, inter-individual variability is important and can be influenced by factors including age, health, and the usage of common medicines like antacids. Protease inhibitors, polyphenols, saponins, phytic acid, and the presence of complex carbohydrates that prevent enzymes from accessing the protein all have an impact on how digestible plant proteins are at this level (114).

Gastric emptying has been the subject of considerable study because it is believed to have several effects related to satiety. Even while eating a small meal can cause the stomach to fill up rapidly, the stomach's release of digesta takes time. Particles larger than 1–2 mm are typically maintained in the stomach until late in the emptying stage due to the sieving effect. Usually, the release of gastric contents happens over a few min to up to 6 h or more, with the primary peak of release occurring after 1.5 to 2 h. Overweight people are said to have faster rates of stomach emptying (111). The relationship between enhanced subjective satiety signals in humans and a decreased stomach-emptying rate, or prolonged gastric residence time, has been established. It has been demonstrated that diets with the same macronutrient composition, whether they are solid or liquid, affect stomach emptying and the release of satiety hormones in the intestines differently. For example, in a study using liquid and gelled lipid-protein emulsions, it was discovered that the liquid diet caused a faster release of nutrients into the lumen, leading to a more rapid nutrient sensing at the proximal part of the small intestine because higher levels of the gastric inhibitory polypeptide (GIP) were discovered in the plasma of liquid-diet-fed rats (114).

This is because food deconstruction and rearrangement during gastric passage have a huge impact on how nutrients are absorbed later on and how full you feel. Food disintegration, viscosity changes, nutrient redistribution, and gelation are all effects of intragastric food structure that can affect gastric distention and emptiness and consequently, satiation and satiety (112).

### Food-related elements

#### Sensory attributes

The sensory characteristics of food play a vital role in the regulation of food intake. Mostly, the appearance of food influences eating, which resultantly governs the amount of food to be consumed. Previously, the potential impact of sensory attributes of food including its appearance, odor, taste, and texture on satiety and satiation has been documented (16). Food odors have been found to either increase or decrease food intake, especially based on individual perceptions. Individual preferences for different odors mainly affect the palatability of foods (115). The palatability of food thus affects the eating process to a great extent as positive hedonic signals before meal initiation can enhance food consumption (16). The impact of food labels indicating a food's satiating attributes has also received less attention. Since expected satiation has been demonstrated to affect hunger ratings and food intake, such labeling may have an impact on how much food is consumed. As this mechanism may be involved in the impact of satiation labels on intake, Hendriks-Hartensveld et al. (116) observed that the effects of such labeling on the magnitude of sensory-specific satiety are a relative decline in the pleasantness of food during consumption experienced after eating the meal.

#### Food structure

Understanding the role of food structure in satiation and satiety becomes tougher as hunger and fullness are influenced by physiological, psychological, and other physical factors before, during, and after the consumption of food. However, evidence suggests that the texture of food is an important element in the arousal of hunger sensations as it directly or indirectly influences oral processing factors such as mastication, chewing efficiency, orosensory time, and self-textural perceptions. Therefore, the texture of food not only determines the overall acceptability of a meal but also influences the satiating potential to a certain degree. The involvement of certain neurons in assessing the orosensory cues may trigger the varying palatability responses for different meals since mouthfeel differs corresponding to the texture of the food which in turn affects satiation and satiety (17).

The physical and rheological properties of foods (solid or liquid) are thought to have an influence on energy consumption owing to their perceived satiating effect (117). The impact of food consistency i.e., raw, semisolid, fluid, or pureed on satiety has already been investigated (118). The first systematic review and meta-analyses on the influences of food texture (form, viscosity, structural complexity) on satiety were presented by Stribitcaia et al. (20). Results delineated that as compared to liquid and low viscous food, both solid and more viscous food reduce hunger. It was also observed that there was an association between viscosity and fullness as well as a moderate relationship between food form and food consumption was also noted. Highly viscous liquids provide more satiety as compared to less viscous liquids. This phenomenon might be explained by the decreased eating rate since a spoon or straw is required coupled with increased engagement of muscle and tongue. As a result, the oral processing time of food is increased, affecting the psychological and physiological signals that control satiety (119).

Food macrostructure usually affects gastric retention, rate of gastric emptying, and nutrient absorption. Purposely, a study was carried out on 10 healthy volunteers to examine the impact of gastric retention on appetite sensations using isocaloric test meals. The results revealed increased gastric retention and a decreased appetite for a semi-solid meal as compared to a liquid intake. This in turn translated into differences in blood glucose and insulin responses that affect satiation and satiety. The increased viscosity in the stomach and improved sensation of intestinal nutrients leads to good appetite control (120). Moreover, the potential of food microstructure in altering satiation response may be elucidated during digestion which is strongly affected by variable particle sizes of the meal. Likewise, the effect of oil droplet size while consuming 2 mm or 50 mm in an emulsion preload suggested that not only perceived liking for creaminess affect appetite but smaller droplet sizes resulted in decreased food intake at subsequent lunch (121). In addition, compared to the milled rye kernel porridge breakfast, satiety was increased, and appetite was suppressed in the afternoon following the ingestion of the rye kernel breakfast. This influence may be attributed to structural variations alone, as the nutritional quality of both commodities was similar, including the content and structure of dietary fibers (122).

Processing also influenced the food structure and often increased the digestibility of foods when compared to raw foods. Resultantly, processing improves glucose availability and is more likely to affect satiation than satiety (123). It is pertinent to mention that unprocessed or raw foods render satiety due to prolonged gastric transit time. The findings of the study explicated that whole apple particularly reduced energy intake from the test meal. Similarly, the effect of instant oatmeal and ready-to-eat oatmeal breakfast on satiety was investigated, the energy intake was particularly reduced after consumption of instant oatmeal in contrast to ready-to-eat oatmeal cereal (124).

#### Portion sizes

Portion size is an important consideration in designing a healthy menu for obese patients. Usually, obese individuals tend to eat more food when offered in large portions size. Many social and cultural norms also promote larger portion sizes that in turn lead to overeating and obesity. Perceived satiation and satiety relative to portion sizes depend upon the volume of the foods (125). The effect of iso-caloric portions of seven different types of bread varying in nutrient composition and physical appearance was assessed for the feeling of fullness scores. Satiety index scores for regular white bread were found to be the lowest, without revealing any correlation between satiety and glycemic response. Besides, less energy intake at test meals was found to be associated with the participant's perceived satisfaction with larger portion sizes. There is a strong link between portion sizes and expected satiety as individual liking serves as a constant stimulus to drive the satiation and satiety sensations (126).

Portion sizes of several convenience food items have tended to gradually increase. The trend has now become common in various settings including supermarkets, restaurants, and homes. This increase in portion size is one of the major causes of the current obesity epidemic. Therefore, choosing a small portion size with a relatively lower energy density is effective in weight management programs. Conversely, sustained consumption of increased portion size can particularly enhance the energy intake which leads to increased body weight (127).

The sensitivity to portion size also differs with age since children < 3 years of age consume a constant amount of food irrespective of portion size as they are more sensitive to essential mechanisms of satiation or satiety. Though, when the large portion sizes were served to 5 years old children, energy intake was significantly increased due to environmental cues acquired with the growing age (128). Likewise, an up to 15% increase in energy intake was observed when 4 years old children were served with double portion sizes (129).

Currently, USDA's recommendation to control portion size and increase smart food choices includes the implementation of USDA's MyPlate. The USDA suggests filling half of your plate with fruits and vegetables, one quarter with grains (half of which should be whole), and one quarter with protein, along with a portion of low-fat or fat-free dairy (130). Calorie restriction, and portion control methods have long been used in primary care-based obesity management. The MyPlate-based obesity treatment strategy, in contrast, promotes consuming more high-satiety/high-satiation foods and does not require calorie counting (131).

#### Energy density

The energy density of the food plays a key role in energy consumption as satiating efficiency is largely affected by energy density (19). The energy density (kJ or kcal/g) denotes the amount of energy available in a given amount of food. The energy density is governed by the food composition since foods rich in fat are energy-dense when compared to those having a significant amount of fiber. Replacement of fat-rich foods with less energy-dense foods enriched with fiber can significantly lower energy intake (19). Food with low energy density tends to increase satiety, suppress hunger, and lessen energy intake. Hence foods with low energy density resulted in a better fullness sensation. Another work revealed that devouring a large portion size and having low energy density increased the average eating time by 33%, improved the satiety response, and displaced energy intake for the subsequent meals of the day (132).

#### Food macronutrients

Among major macronutrients, the protein content of the food significantly affects the satiety value when compared to fats and carbohydrates (133). Apart from proteins, soluble fiber is the other promising ingredient with a high satiating ability. Although attributing satiety to a single factor is not very meaningful, a variety of food attributes including structure, complexity, composition, etc., often act in combination at more than one level.

##### Carbohydrates

Carbohydrates are a diverse group of biomolecules consisting of a single (monosaccharides), two (disaccharides) few (oligosaccharides), and multiple monomers (polysaccharides). The impact of carbohydrates on satiation and satiety primarily depends upon their digestion, absorption, and metabolism, since long-chained polymers take more time for digestion when compared to sugars. Thus, changes in the level of blood glucose and satiety hormones (insulin and amylin) are attributed to a variable rate of carbohydrate metabolism. The decline in food intake after consuming carbohydrates is often associated with sensory stimulation, gastric distention, and nutrient intestinal contact (134). Hence, satiety from carbohydrates relies on the form in which it is delivered.

Considering the short-term effects of carbohydrates on satiety, individual sugars may also have a variable response, since the ingestion of glucose instantly increases the blood glucose and insulin levels in contrast to sucrose. Fructose has the least effect on blood glycemic response. Fructose also improved satiety, but the relative impact of preloads significantly controlled the food intake, since no difference in food intake was observed between 50 g fructose and 50 g glucose at 2.25 h when they were given in a mixed nutrient meal containing starch (135). Thus, the changes in blood glucose after ingestion of different sugars and subsequent decline in food intake conform well with the Glucostatic Theory presented by Mayer in 1953 which states that the onset of feeding occurs upon low blood glucose level while increased glucose level suppresses the food ingestion and governs satiation (136).

Apart from sugars, work has been conducted on the relationship between the glycemic index (GI) of foods and satiety. GI represents the increase in blood glucose in response to carbohydrate-containing foods. There is an inverse relationship between the satiety value of different foods and their GI. In this perspective, a study found that appetite and food intake were significantly suppressed on ingestion of high-GI foods as long as high blood glucose levels were persistent (137). In short-term satiety, a sudden rise in blood glucose occurs on the consumption of high GI foods, but in the case of long-term satiety, consuming low GI foods leads to a slow and steady release of glucose that helps to sustain euglycemia with improved appetite sensations. A satisfactory satiety response can be achieved using low GI diets with the same energy density. Thus, a diet with low GI and reduced energy content can be beneficial to shedding excess body weight by controlling glucose metabolism and insulin response (138).

##### Dietary proteins

Protein is a strong determinant of satiety as multiple investigations have validated the hypothesis that high protein diets provide an enhanced feeling of fullness. Increased protein content in the diet may result in increased thermogenesis and energy expenditure due to a strong thermic effect. Protein-rich diets elicit increased satiety as their metabolism leads to a greater number of amino acids escaping the protein synthesis channel and reaching the blood plasma, thereby serving as a satiety signal to suppress further food intake (139). Different mechanisms are involved in the satiety regulation after ingestion of protein including increased productions of satiety-related hormones i.e., PYY, glucagon-like peptide-1, and cholecystokinin coupled with a lower level of orexigenic hormone-ghrelin. It is noteworthy that not only protein-enriched diets but also isolated proteins like whey and casein have a significant effect on satiety and retain discrete satiety mechanisms (140, 141). The casein fraction of milk proteins is one such example that delays gastric emptying by getting coagulated in the acidic environment of the stomach. Unlike casein, whey proteins remain soluble at the gastric pH, rapidly passing through the stomach and resulting in faster absorption of amino acid and subsequent metabolic response. Therefore, less release of GLP-1 was observed after casein intake in contrast to whey protein, thereby promoting satiation (114).

The meta-analysis by Yang et al. (142) to compare protein-rich vs. normal protein diets has been conducted to assess postprandial satiety response. Results demonstrated that acute high protein intake (>20 % of energy from protein) did increase satiety and have a higher thermogenic effect with moderate heterogeneity between studies. Additionally, compared to normal protein test meals, high protein test meals may help control postprandial glucose. Likewise, a study was conducted to compare the effects of different proteins such as whey with or without glycomacropeptide (GMP), casein, and soy proteins. Satiety was higher after casein or soy-based high-protein meals and lower after whey-GMP-based high-protein breakfasts. Though high protein breakfast with whey and GMP satiety results due to an increase in GLP-1 (satiety hormone) (140). Another important feature of a high protein diet is an amino acid-induced increase in gluconeogenesis which may contribute to protein-induced satiety. Such an effect of a high protein diet on gluconeogenesis has been studied previously. The results revealed enhanced gluconeogenesis after the consumption of a high-protein diet. As a study carried out on appetite control drew a similar conclusion where decreased food intake was associated with high protein foods when the subjects received an isoenergetic high-protein diet (30, 0, 70% energy from protein, carbohydrate and fat) or normal-protein diet (12, 55 and 33% energy from protein/carbohydrate/fat) in a randomized cross-over design (143).

Furthermore, the comparison between animal and plant protein on satiety and glucose response in an iso-caloric breakfast revealed the usefulness of animal protein in regulating postprandial glucose response and satiety (144). Among animal proteins, eggs possess the greater potential to delay hunger as well as contain many other beneficial macros and micronutrients essential for health maintenance (145). Likewise, no difference was recorded in the satiating response of fish and beef protein (146). However, a significant decline in energy intake was observed at the subsequent meal after the consumption of fish. This decline was attributed to the slow digestion of fish, owing to specific amino acid profiles. Therefore, varying the protein sources in a mixed meal may play a significant role in metabolic kinetics. The insulin, glucose, and glucagon responses vary owing to the difference in the gastric emptying rate of various proteins (casein, gelatin, soy protein), that in a turn depends on the amino acid profiles (147).

There is consistent evidence that protein in an adequate dose has more impact on satiety as compared to corresponding amounts of carbohydrates or fat. This has also been confirmed by long-term weight loss studies, which showed that a high-protein diet was more effective in eliciting a satiety response than a low-protein diet, thus helping in promoting weight loss by reducing the amount of food intake (148). This was probably due to the greater satiety effect of protein as compared to fats and carbohydrates. However, variations in study designs cause difficulty in assessing the optimum protein dose or energy share required to detect the noticeable effects on satiety. Usually, at least 50 g of protein in each meal has been suggested to get any substantial effect on satiety, but not enough data is available to define a dose-response relationship (149).

##### Dietary fats

The fat-driven satiation effects are mainly induced by triacylglycerol (150) and free fatty acids. The dietary fats i.e., saturated, monounsaturated, and polyunsaturated fatty acids can be detected by the lingual lipase (upon fatty acids stimulus). Purposely, fatty acid receptors namely GPR120 and GPR40 sense the intake of dietary fat in the gut. In response to fat intake, a gut peptide released lead to altered gastrointestinal tract movement. Intestinal beta-oxidation of fatty acids is carried out through fatty acid transporter CD36, protein kinase C-zeta, protein kinase C-delta, and the 2-monoacylglycerol receptor GPR119 (151). The mechanism of appetite control and intake of fat energy vary (152). Enterocytes release a satiety signal called oleoyl ethanolamide (OEA) having an anorexigenic effect which acts on intestinal receptor PPAR alpha through vagal afferent nerves. Accordingly, the c-fos region of the brain, hypothalamic paraventricular (PVN) area, a nucleus of the solitary tract and supraoptic nuclei (SON) are activated thereby regulating food intake (153). Although a decrease in energy intake following the consumption of a high-fat diet (due to a lower amount of food eaten) has been observed, short-term studies suggest that ingestion of fat reduces not only eating time but the sensation of hunger as well, thus promoting satiation in contrast to satiety (154). Long-term studies are required to explain this increase in energy intake attributed to a variable mechanism of appetite regulation for a high-fat diet. Although some short-term studies (2–3 weeks) reported an effect of a high-fat diet on appetite suppression, however doubts were cast on their analytical approach. The high-fat diet altered the ability of the GI tract to sense fat and resulted in an enhanced energy intake. Such mechanisms have now become an important part of research to treat obesity (152).

The effect of dietary fatty acids on satiety revealed that the response of PYY was significantly lower with meals high in monounsaturated fatty acids when compared to meals enriched with polyunsaturated and saturated fatty acids (155). It is pertinent to mention that PYY is a hormone secreted by the gastrointestinal tract (GIT) to inhibit the orexigenic neuron's response to enhance satiety. Similarly, the satiation effect of medium-chain triglycerides and long-chain triglycerides is more pronounced owing to the greater post-meal oxidation of fats (156). Oxidation of fatty acids in plasma is dependent upon the concentration of glucose in the blood since insulin not only governs glucose uptake but is also involved in lipogenesis (157). Erstwhile, medium-chain triglycerides were found to be more satiating as compared to short-chain fatty acids, conjugated linoleic acid, n-3 polyunsaturated fatty acids, diacylglycerol, and small particle lipids. Such an effect was attributed to either fatty acid oxidation that enhanced ketone bodies i.e., β-hydroxybutyrate or the release of anorexigenic hormones CCK or PYY that require fatty acids with chain lengths of 12 and above to accomplish this effect (158).

##### Dietary fiber

The dietary fiber provides satisfaction and satiety by adding bulk and increasing the viscosity of the digesta along with GIT. The non-availability of valid biomarkers of fiber functionality related to satiety makes it difficult to compare dietary fibers for their role in satiety. Many functional fibers (inulin oligofructose, polydextrose, and resistant starch) that are not viscous have little or no effect on satiety. While other functional fibers, mostly viscous (pectin, psyllium, and guar gum) or microbiological produced (xanthan gum or pectin) increased satiety (1). Even if several studies have indicated a decrease gastric-emptying rate after viscous fibers intake i.e., pectin (159, 160), guar gum (161), β-glucan (162), and alginate (163), other opposite results showed no such effects (164–166), further research is needed for clarifying these fibers effects on energy balance and satiety, including those on the related mechanisms (167). Pectin is prebiotic with health-promoting effects, such as regulation of glucose homeostasis and lipid metabolism, and other potential health effects poorly understood until now, including obesity prevention (168). The effect of pectin is associated with improvements in insulin and glucose profiles (169, 170), and also with influences on leptin and adiponectin circulating levels, thus resulting in a decrease leptin/adiponectin ratio. Besides, the high-esterified pectin (HEP), which can be found in vegetables and fruits, is fermented more slowly in GIT in comparison with that low-esterified, the complete fermentation being carried out probably in the colon, which shows a larger and a higher variety of bacterial microflora (171, 172), thus showing a higher inhibition of glucose absorption at the intestinal level, and improved insulin resistance and of other factors related to cardiovascular health (173). HEP is a major component of soluble dietary fiber, with potentially benefic effects on metabolic disorders and obesity, showing associations with health-promoting effects related to body weight, glucose homeostasis, and lipid metabolism, even that the explanation of these benefits is not clear if it resides in the calorie intake decrement or other unveiled mechanisms (21). Moreover, HEP supplementation is able to modulate, in terms of restoring or improving, leptin/adiponectin signaling pathway and lipid metabolism throughout the oxidative/lipogenic balance in liver, being also associated with insulin and leptin sensitivity improvements, not specifically attributed to a decrease in energy intake, but to other mechanisms involved (168). Related to β-glucans, short-term and long-term studies assessed the effect of oat β-glucans in transforming diet, indicating its ghrelin, PYY, GLP-1, GIP and leptin modulating abilities (174). Besides, the oat β-glucan dietary supplementation in patients with type 2 diabetes showed effects such as improved glycemic control e.g., higher insulin secretion, but no significant differences in leptin and ghrelin, with an increase in GLP-1 and PYY that showed increased satiety perception and modified gut microbiota having healthier profile (174), contrary to other scientific reports (175). Like guar gum, xanthan results in slower gastric emptying of glucose and nutrient energy and shows resistance to bacterial breakdown, thereby its supplementation adding little, short-chain fatty acid *via* its bacterial decomposition in the gut. From the earlier reports indicating the potential of xanthan gum to be used in the dietary management of diabetes mellitus (176) and its effect on satiety in obese patients after test meal (177), recent studies conducted on the potential of using xanthan gum in emulsions. Interestingly, even if it shows lower viscoelasticity in water solution after stomach incubation, due to the reduced electrostatic repulsion in the acidic environment, thus driving to more flexible chains, on the opposite, the xanthan gum emulsion has higher viscoelasticity in the stomach based on the fat coalescence and coagulation induced by the weakness of its supporting structure (178). Whole foods consumption and their effects on satiety depend upon the kind of dietary fiber present, their viscosity as well as gut microbiota. A decrease in appetite by dietary fibers from sources like barley and oats is well reported (124). Apart from increased viscosity, β-glucan from oats imparts satiety by the increased postprandial release of cholecystokinin (124). Likewise, poor appetite ratings after consumption of wheat bran and psyllium husk had been attributed to increased viscosity and solubility of the fibers (179). Difference in insoluble and soluble dietary fiber induced satiety is subtle due to difference in action during consumption (satiation) and following consumption (satiety). In trials investigating non-viscous soluble fibers such as inulin and resistant starch, non-significant effect on satiety was witnessed. The fat content of a diet may be able to influence the total energy intake, thus, reducing dietary fats could drive to a lower total energy intake and a decreased weight gain, such statements being supported by many investigation trials. Even so, dietary fats effect on energy intake needs further assessment for clarifying if it is due to only its higher energy density or if it shows impact unrelated to energy density. Moreover, the satiety value for non-fermentable fiber is higher as compared to the fermentable ones (1). Consuming dietary fiber on a daily basis mainly in the form of salad can remarkably reduce the energy intake. Women ate pasta as a main course *ad libitum* on five different occasions, four times with a low-energy-dense salad (300 g, 100 kcal). The salad was provided 20 min before the pasta at two meals (once mandatory; once *ad libitum*), and the salad was presented with the pasta at two meals (once compulsory; once *ad libitum*). According to the findings, including a set amount of salad in the meal lowered energy intake by 11%, while eating a low-energy-dense salad before the main course increased vegetable consumption by 23%. Further results revealed that such an effect was correlated with serving size but was independent of the timing of intake (180).

#### Bioactive compounds

Certain bioactive ingredients in food can also influence satiety and subsequent energy intake. For example, caffeine has been found to influence energy balance as its prolonged consumption may help in weight loss. Similarly, the consumption of beverages containing caffeine or catechins in the form of green tea delays hungers arousal, thereby reducing energy intake (181). Likewise, the effects of capsaicin, green tea, and sweet pepper on hunger and appetite sensations along with energy intake have also been studied. The results revealed that a combination of green tea and capsaicin can effectively reduce energy intake in negative energy balance by enhancing satiety and suppressing hunger (182). In another study, a positive correlation between capsaicin and satiety has been found due to the release of satiety hormones (183).

#### Functional foods

The urge to discuss satiety and appetite regulation seems to be more than ever. Food manufacturers are always looking to provide items that people would be more willing to consume. Their goal is to provide goods that increase consumer appetite. Therefore, the increasing incidence of obesity and overweight issues is always attributed to the food industry (4). As a result, many food manufacturers throughout the world are changing the formulation of their products to develop products that can decrease appetite and calorie consumption, particularly in obese and overweight persons (184). Introducing functional foods in the market to suppress appetite requires consideration of crucial factors: efficacy, feasibility, acceptability and effective size (185). Some substances have an indirect effect on appetite while each product must also be feasible in terms of the equipment needed for production, processing, and storage. Additionally, when designing such products, consideration must be given to the magnitude of each compound or the total of compounds' effects on hunger (186). Functional food is included in products that make performance-related claims and claims to decrease appetite. In this way, they influence the body's function or feeling of appetite and may modulate it. Most producers frequently misuse these items, which leads to consumer confusion. Any claim of reduced appetite must be supported by credible, scientific evidence. Long-term human studies should support any claims of weight loss that may follow from using appetite suppressants. Any claim that a substance decreases appetite should also be presented in comparison. As a result, two groups—one control and one intervention must be chosen, their respective levels of appetite reduction must be assessed, and confounding variables must be taken into account (187). The price of proteins and fibers is typically substantially greater than that of other ingredients used in the food industry, and they are typically among the key components of most products planned and produced to lower hunger. Such products will cost more since a combination of vitamins and minerals will be added to them to prevent malnutrition. Therefore, it can be acknowledged that the people or groups with high social and economic standing are the target market for the majority of functional foods, which is seen as one of their limitations (188).

## Satiety response of food groups

Food groups such as cereals, meat, fat, fruits and vegetables, and dairy products (Table 1) vary in their ability to satisfy hunger as there are multiple putative mechanisms by which food components send signals to the brain, which affect the gut and induce satiety.

**Table 1:** Summarizing results of studies assessing variation in satiety among food groups.

**Food groups**	**Aim of the study**	**Foods tested**	**Satiety measurement**	**Results**	**References**
Cereals	Effect of two oat-based cereals on subjective ratings of appetite	Two oat-based ready-to-eat cereals; RTEC1: Quaker Oatmeal Squares and RTEC2: honey nut cheerios	100 mm visual analog scale	Similar amounts of oat β-glucan in products but different functionality was observed as more fullness or desire to eat found after RTEC1	(189)
	Effect of rye bread breakfasts on subjective hunger and satiety	Rye bran bread, intermediate rye fraction bread, Sifted rye flour bread and wheat reference bread	100 mm visual analog scale	Significant results for rye bread in reducing appetite sensations	(190)
	Variation in satiety for cooked Philippine rice having a different glycemic index	Seven rice varieties	Satiety Quotient	Variation in satiety scores was associated with dry matter content of rice	(191)
	Effect of whole meal pasta on subjective satiety and plasma PYY concentration	Wholemeal pasta and refined wheat pasta	Visual analog scale GLP-1, ghrelin, PYY	Whole grains control the appetite instead of refined wheat pasta	(192)
	Satiety from rice-based, wheat-based and rice–pulse combination preparations	Reference bread, Semolina preparation, “Upma” broken wheat preparation, “Dalia upma”, whole wheat flat bread, “Paratha” and rice flakes preparation, “Poha” Fermented rice–pulse preparation, “Idli”	100 mm visual analog scale	Fermented rice pulse combination exhibited the highest satiety scores	(193)
	Wholegrain vs. refined wheat bread and pasta. Effect on postprandial glycemia, appetite, and subsequent *ad libitum* energy intake	Refined wheat bread, wholegrain wheat bread, refined wheat pasta and wholegrain wheat pasta	100 mm visual analog scale	Whole grain wheat bread resulted in increased satiety and fullness compared to the refined wheat bread	(194)
	Effect of biscuits formulated with high-amylose maize flour on satiety	Control biscuits of commercial white wheat flour and biscuits made from corn containing 25 and 50% amylose	10 cm Visual Analog Scale	Increasing the level of wheat starch substitution with maize flour up to 50% resulted in a greater reduction in food intake at a subsequent meal	(195)
Meat and meat products	Effect of different textures of foods on satiation	Meat and meat replacer	100 mm visual analog scale	The negligible difference found for fullness and prospective consumption	(196)
	Acute satiety response and hormonal markers of appetite after consuming different types of meat	Chicken, pork and beef	Blood biomarkers; Ghrelin, PYY, Insulin, Glucose and CCK	Equated results for satiety response upon pork, beef, and chicken ingestion	(197)
	Postprandial glycemic and satiety response for fish protein hydrolysate in healthy adults	Boarfish protein hydrolysate (BPH) drink	Visual analog scale Ghrelin and leptin	No significant effect on biomarkers of satiety	(198)
	Effects of a beef-based meal compared to a calorie matched bean-based meal on appetite and food intake	Beef and beans	Visual analog scale	Beef-based meal with high protein and a bean-based meal with moderate protein and high fiber produced similar satiety	(199)
Fats and oils	Effect of fat saturation on satiety, hormone release, and food intake	Shea oil, canola oil and safflower oil	Visual analog scale	Triacylglycerols with unsaturated fatty acids increase satiety than with saturated fatty acids	(200)
	Coconut oil has less satiating properties than medium-chain triglyceride oil (MCT oil)	MCT oil, coconut oil and vegetable oil	Visual analog scale	MCT also increased fullness over the 3 h after breakfast compared to the vegetable and coconut oils	(201)
	The gastric emptying rate for specific food structures and impact on appetite suppression	Control meal (an emulsion of sunflower oil) and structured/active meal (gouda cheese and low-fat yogurt)	Visual analog scale	Active or structured meal significantly reduces hunger	(120)
	Effect of fat source on satiety	Canola and peanut oil muffins and canola, peanut oil, butter muffins	nine-point category scale	The slightly different satiating effect between saturated and monounsaturated fatty acids	(202)
	Effect of replacing breakfast with a high-fat drink	High fat (medium-chain triglycerides) meal replacement drink	Satiety labeled intensity magnitude	Increased satiety was reported in the afternoon after a high-fat meal replacement drink	(203)
Fruits and vegetables	Appetitive responses in lean and obese adults after ingesting fruits in solid vs. beverage forms	Solid fruit preload (red seedless grapes, dried apples, gala apple, raisins) and beverage fruit juice preloads	nine-point scale	Delaying time for hunger arousal was higher for solid fruit preloads	(40)
	Subjective assessment of hunger and fullness in healthy adults after ingesting orange pomace	Whole orange fruit, orange pomace and orange juice	Visual analog scale	The addition of orange pomace fiber to orange juice and whole fruit increased satiety in orange juice	(204)
	Using avocado as a test meal to test satiety	Whole avocado	Visual analog scale	Avocado-derived fat-fiber combination increased feelings of satiety and anorexigenic hormones PYY and GLP-1	(205)
	The effects of wild blueberries on satiety and glycemic control	Blueberry, blueberry juice, placebo beverage and control	Visual analog scale	Higher satisfaction when the whole blueberry treatment was consumed compared to the control	(206)
	Effects of Fresh Watermelon Consumption on the Acute Satiety Response	Watermelon and low-fat cookies as control snack	Visual analog scale and appetite regulating hormones	watermelon elicited robust satiety responses than cookies snacks. Watermelon also resulted in reduced leptin hormone and higher ghrelin	(207)
	Effect of fresh mango consumption on satiety	Fresh mango and iso-caloric low-fat cookies as control	Visual analog scale and appetite regulating hormones	Mangoes promote greater satiety and cookies did not reduce participants' desire to eat	(208)
	Influence of dietary carbohydrates and glycaemic response on subjective appetite and food intake	Potato, barley, glucose and placebo	Visual analog scale	Potatoes increased subjective satiety the most, followed by barley, then glucose	(209)
	Comparison of low glycemic index and high glycemic index potatoes about satiety in humans	Carisma® low Glycemic Index potatoes and Arizona high Glycemic Index potato varieties	Visual analog scale	No significant differences in the primary endpoint, satiety	(210)
	Effect of fenugreek fiber on satiety, blood glucose and insulin response	0, 4 and 8 g fenugreek extract beverage	Visual analog scale	Fenugreek fiber (8 g) significantly increased satiety	(211)
	Effect of capsaicin on satiety and energy intake	0.9 g of red Pepper in tomato juice, 0.9 g of red pepper in two capsules	Visual analog scale	The AUC for satiety increased, whereas the AUC for hunger decreased after capsaicin ingestion	(212)
	The effects of the fiber content and physical structure of carrots on satiety and subsequent intakes when eaten as part of a mixed meal	Whole carrots, blended carrots and carrot nutrients	Visual analog scale	Meals with whole carrots and blended carrots resulted in significantly higher satiety	(213)
Milk and milk products	The satiating potential of yogurt enriched with protein	Yogurt products	nine-point scale	Highest satiety scores for yogurt having added milk proteins	(214)
	Satiety and food intake after consuming different dairy products	Milk products	Visual analog scale	An increase in satiety has been observed after 500 ml of milk	(215)
	Effects of goat dairy and cow dairy-based breakfasts on satiety	Goat or cow dairy breakfast	Visual analog scale	The slightly higher satiating effect of goat dairy when compared to cow dairy	(216)
	Effect of casein-to-whey ratio in breakfast meals on postprandial satiety ratings	Milk with 80:20 or 40:60 casein-to-whey protein ratios	100 mm visual analog scale	The protein ratio did not significantly differ in satiety ratings after the second meal	(217)
	Satiety response of milk protein-derived peptides	Milk protein-derived peptides; sodium caseinate and a whey protein hydrolysate	Cumulative food intake	Sodium caseinate derived peptides suppressed appetite more than other peptides.	(218)
	Effects of cultured dairy and non-dairy products added to breakfast cereals on blood glucose control, satiation, satiety, and short-term food intake	Greek yogurt with granola, cultured coconut product with granola and water	100 mm visual analog scale	Intake of dairy suppresses the mean 2-h subjective appetite stronger compared to the non-dairy	(219)
	Milk protein fractions moderately extend the duration of satiety compared with carbohydrates	Milk proteins; casein, whey and their mixture	Energy intake at lunch	Compared with the control snack, proteins extended the duration of satiety with no difference between the protein groups	(220)
	Effects of low-fat milk consumption at breakfast on satiety and short-term energy intake	Low-fat milk, apple juice and water with breakfast	Visual analog scale	Obese children reported higher satiety score after drinking low-fat milk with breakfast	(221)

Variation in satiety responses among these five basic food groups exists since they offer different macronutrient compositions as cereals are high in carbohydrates, while meat and meat products are rich sources of proteins. Likewise, fruits and vegetables provide soluble and insoluble dietary fiber. Apart, satiety index scores for a variety of isocaloric foods have also been developed (27). Among all food groups, fruits and vegetables received the highest satiety scores, and refined cereal products gained the lowest satiety scores. Considering many internal and external factors, the food matrix may particularly affect satiation and satiety due to its interaction with the gut at various levels from ingestion to absorption along with other related components being discussed in the review.

## Conclusions

Satiety is a complex and dynamic process that can be modulated while attempting to achieve improved fullness and reduce caloric intake. Different strategies for individual health goals are often applied to regulate the underlying factors affecting food intake from the cephalic to gastric phase. The meals high in protein, with larger portion sizes and lower calorie density, as well as higher viscosity of digesta (either solid or semisolid), stomach emptying and controlling hedonic hunger improve the satiety response, whereas satiation is enhanced with the high-fat foods. Furthermore, the post-digestive or post-absorptive response of foods greatly affects satiation or satiety through gut-brain signaling and energy homeostasis. Besides, body composition (more leptin in females), specific meal size in different cultural cuisines, increased food mastication, consistent physical activity, and overexpression of anorexigenic hormones triggered by the SCFA produced by the gut microbiome upon dietary fiber consumption are just a few of the personal factors that may lead to reduced food intake or improved satiety signaling. Since eating behaviors are heritable, variations in physical activity, sleep, and circadian rhythm all together play an important role in explicating an individual's food intake patterns. The current review has thus examined the totality of the evidence for several personal and food-related factors that may influence the consumption of foods or in turn satiety eliciting response. However, further interventions focusing on the systemic impact of nutrients (e.g., *via* gut microbiota modulation) need to be designed for a long enough time to better understand nutrient-induced satiety and weight regulation.

## Author contributions

Conceptualization: FM and AR. Investigation: FM and RA. Data curation: MA and FM. Writing—original draft preparation: FM, AR, and MS. Writing—review and editing: FM, WA, AH, CS, and MR. Visualization: AVR, MS, and RA. Provided guidance in the manuscript revision: AVR and RA. All authors have read and agreed to the published version of the manuscript.

## Funding

This work is based upon the work from COST Action 18101SOURDOMICS-Sourdough biotechnology network towards novel, healthier and sustainable food and bioprocesses (https://sourdomics.com/; https://www.cost.eu/actions/CA18101/; https://www.cost.eu/actions/CA18101/), where the authors [CS, ARu, and AH] are [Members] of the Working Groups [7 and 8]. SOURDOMICS supported by COST (European Cooperation in Science and Technology). COST is a funding agency for research and innovation networks. COST Actions help connect research initiatives across Europe and enable scientists to grow their ideas by sharing them with their peers - thus boosting their research, career and innovation. This work was also supported by a grant from the Romanian National Authority for Scientific Research and Innovation, CNCS-UEFISCDI, project number PN-III-P2-2.1-PED-2019-1723 and PFE 14, within PNCDI III.

## Conflict of interest

The authors declare that the research was conducted in the absence of any commercial or financial relationships that could be construed as a potential conflict of interest.

## Publisher's note

All claims expressed in this article are solely those of the authors and do not necessarily represent those of their affiliated organizations, or those of the publisher, the editors and the reviewers. Any product that may be evaluated in this article, or claim that may be made by its manufacturer, is not guaranteed or endorsed by the publisher.
